# Primary Leptomeningeal Lymphoma Presenting As Rapidly Progressive Hydrocephalus: Diagnosis and Symptom Relief Through Endoscopic Ependymal Biopsy and Third Ventriculostomy

**DOI:** 10.7759/cureus.78611

**Published:** 2025-02-06

**Authors:** Shota Yamashita, Michiko Yokosawa, Yoji Yamashita, Naomi Sato, Tsutomu Sakuma, Kunihiko Umezawa, Hidenori Endo

**Affiliations:** 1 Department of Neurosurgery, Iwate Prefectural Central Hospital, Morioka, JPN; 2 Department of Neurosurgery, Miyagi Cancer Center, Natori, JPN; 3 Department of Pathology, Iwate Prefectural Central Hospital, Morioka, JPN; 4 Department of Neurosurgery, Tohoku University Graduate School of Medicine, Sendai, JPN

**Keywords:** endoscopic third ventriculostomy, hydrocephalus, neuroendoscopy, primary central nervous system lymphoma, primary leptomeningeal lymphoma

## Abstract

Primary leptomeningeal lymphoma (PLML) is a rare variant of primary central nervous system lymphoma, distinguished by the absence of parenchymal lesions. We describe an exceptionally rare case of PLML in an 86-year-old woman presenting with hydrocephalus. Magnetic resonance imaging showed ventricular enlargement over a two-week period, accompanied by faint periventricular contrast enhancement. To diagnose and treat her symptoms, an endoscopic ependymal biopsy and third ventriculostomy were performed concurrently, resulting in a pathological diagnosis of diffuse large B-cell lymphoma, specifically PLML, and symptom alleviation. The endoscopic approach proved effective and reasonable for the diagnosis and resolution of this atypical hydrocephalus.

## Introduction

Primary central nervous system lymphoma (PCNSL) is a relatively rare subtype of non-Hodgkin lymphoma, which accounts for approximately 2%-5% of all brain tumors and typically presents as a solitary parenchymal mass [1,2]. The incidence of PCNSL in Japan increased from 3.5% between 2001 and 2004 to 4.9% between 2005 and 2008 [3,4]. Primary leptomeningeal lymphoma (PLML) is an even rarer variant of PCNSL that is characterized by the absence of mesenchymal lesions. The estimated incidence of PLML is reported to be 7% of all PCNSLs [5]. Despite the lack of systematic statistics, a report by Taylor et al. summarizing the largest series of 48 cases indicated progression-free survival of eight months and overall survival of 24 months, suggesting a very poor prognosis [5]. Leptomeningeal or ventricular enhancement on magnetic resonance imaging (MRI) is a characteristic feature, but diagnosing PLML is challenging as it mimics infectious, inflammatory, or other tumorous diseases [6-9]. Moreover, the symptoms of PLML vary depending on the location of the lesion and include cranial neuropathy, leg weakness, headache, ataxia, and others [5].

Here, we describe an exceptionally rare presentation of PLML as hydrocephalus with unusually rapid progression. Although the diagnosis was challenging due to poor positive findings, concurrent endoscopic ependymal biopsy combined with third ventriculostomy were instrumental in achieving both diagnosis and resolution of the hydrocephalus.

## Case presentation

**Figure 1:**
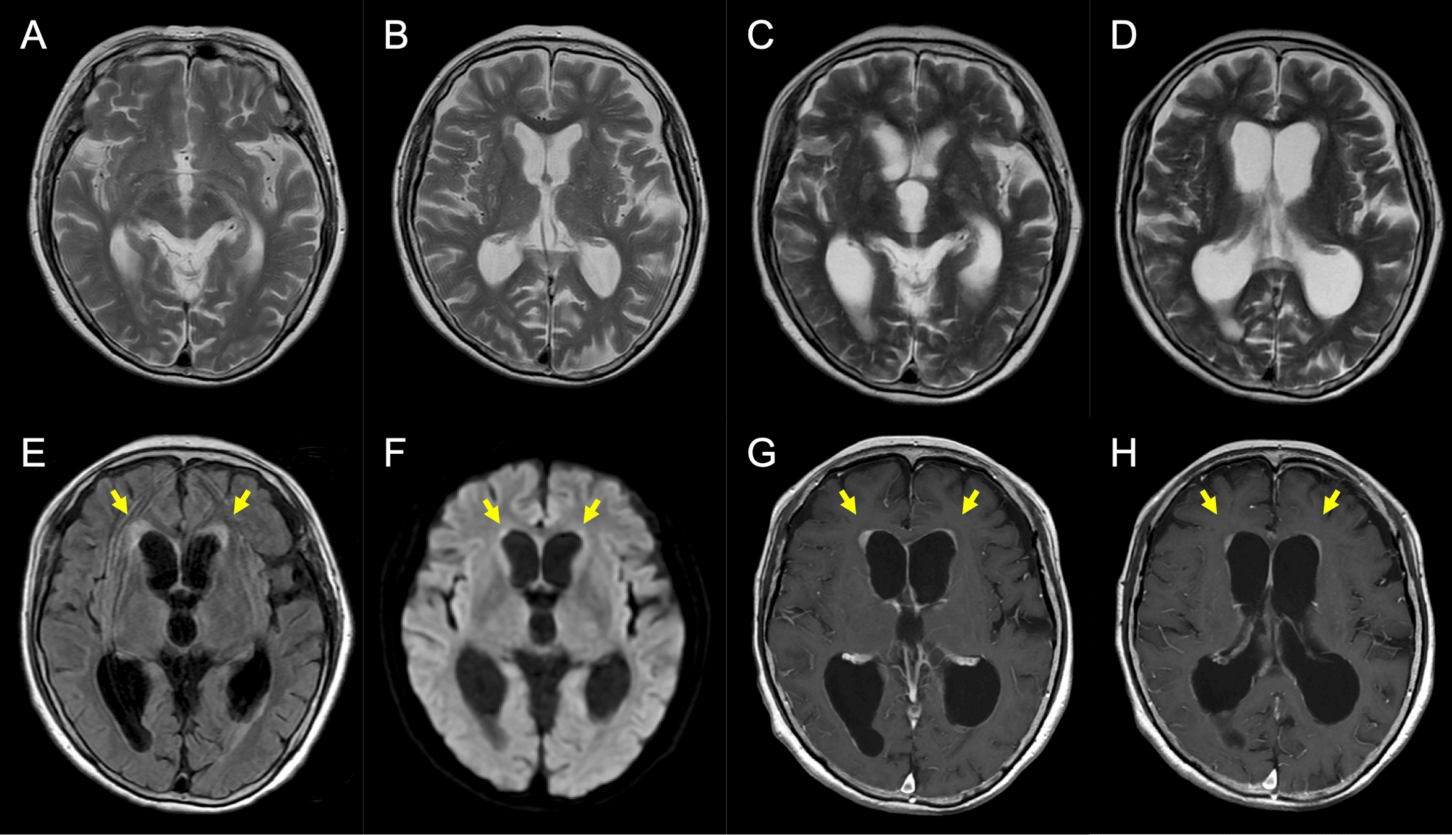
(A,B) T2-weighted images obtained at the local clinic two weeks before the initial visit to our hospital. Sections through the third ventricle (A) and the lateral ventricles (B). (C-H) Magnetic resonance images obtained at our hospital. T2-weighted images (C,D), fluid-attenuated inversion recovery image (E), diffusion-weighted image (F), and contrast-enhanced T1-weighted image (G,H). Each ventricle appears to enlarge in only a two-week period. Yellow arrows indicate abnormal slight hyperintensity areas on fluid-attenuated inversion recovery (E) and diffusion-weighted imaging (F), or faint contrast-enhancement on the T1-weighted image (G, H). Sections through the third ventricle (C,E,F,G) and the lateral ventricles (D,H)

An 86-year-old woman who had fallen and sustained a head injury underwent MRI screening at a local clinic, but no neurological or imaging abnormalities were identified (Figures 1A, 1B). Two weeks later, she returned to the clinic with complaints of gait disturbance and cognitive impairment, at which time marked enlargement of the ventricles was observed. The next day, she visited the Department of Neurology, Iwate Prefectural Central Hospital, for further investigation, including MRI with contrast. The magnetic resonance images revealed proportionally enlarged ventricles with slight hyperintensity at the margins of the anterior horns of the bilateral lateral ventricles on fluid-attenuated inversion recovery and diffusion-weighted imaging, as well as faint enhancement in these regions and no evidence of a mass lesion (Figures 1C-1H), while systematic contrast-enhanced computed tomography screening revealed no abnormal lesions. Cerebrospinal fluid (CSF) analysis showed a normal opening pressure (9 cmH_2_O), pleocytosis with lymphocytic predominance, low glucose, and high protein (Table 1). Both the cytology and bacterial culture were negative. Serum screening for tumor markers, including soluble interleukin-2 receptor, yielded negative results [10]. Based on these results, it was impossible to determine whether the etiology of the hydrocephalus was neoplastic, infectious, or other. The patient's level of consciousness had deteriorated to a somnolent state by the third day of hospitalization (the 18th day after the initial clinic visit).

**Table 1:** Laboratory investigations at admission to our hospital

Parameters	Measured value	Reference range
Cerebrospinal fluid analysis		
Cell count	76/µL	0-5/µL
Mononuclear cells	86%	-
Glucose	26 mg/dL	50-80 mg/dL
Protein	430 mg/dL	10-45 mg/dL
Complete blood count		
White blood cell	6,970 × 10^3^/µL	3.3-8.6 × 10^3^/µL
Red blood cell	3,420 × 10^6^/µL	3.86-4.92 × 10^6^/µL
Hemoglobin	14.1 g/dL	11.6-14.8 g/dL
Platelet	165 × 10^3^/µL	158-348 × 10^3^/µL
Serum analysis		
Albumin	3.9 g/dL	4.1-5.1 g/dL
Lactate dehydrogenase	264 U/L	124-222 U/L
C-reactive protein	<0.03 mg/dL	<0.14 mg/dL
Soluble interleukin-2 receptor	270 U/mL	157-474 U/mL

**Figure 2:**
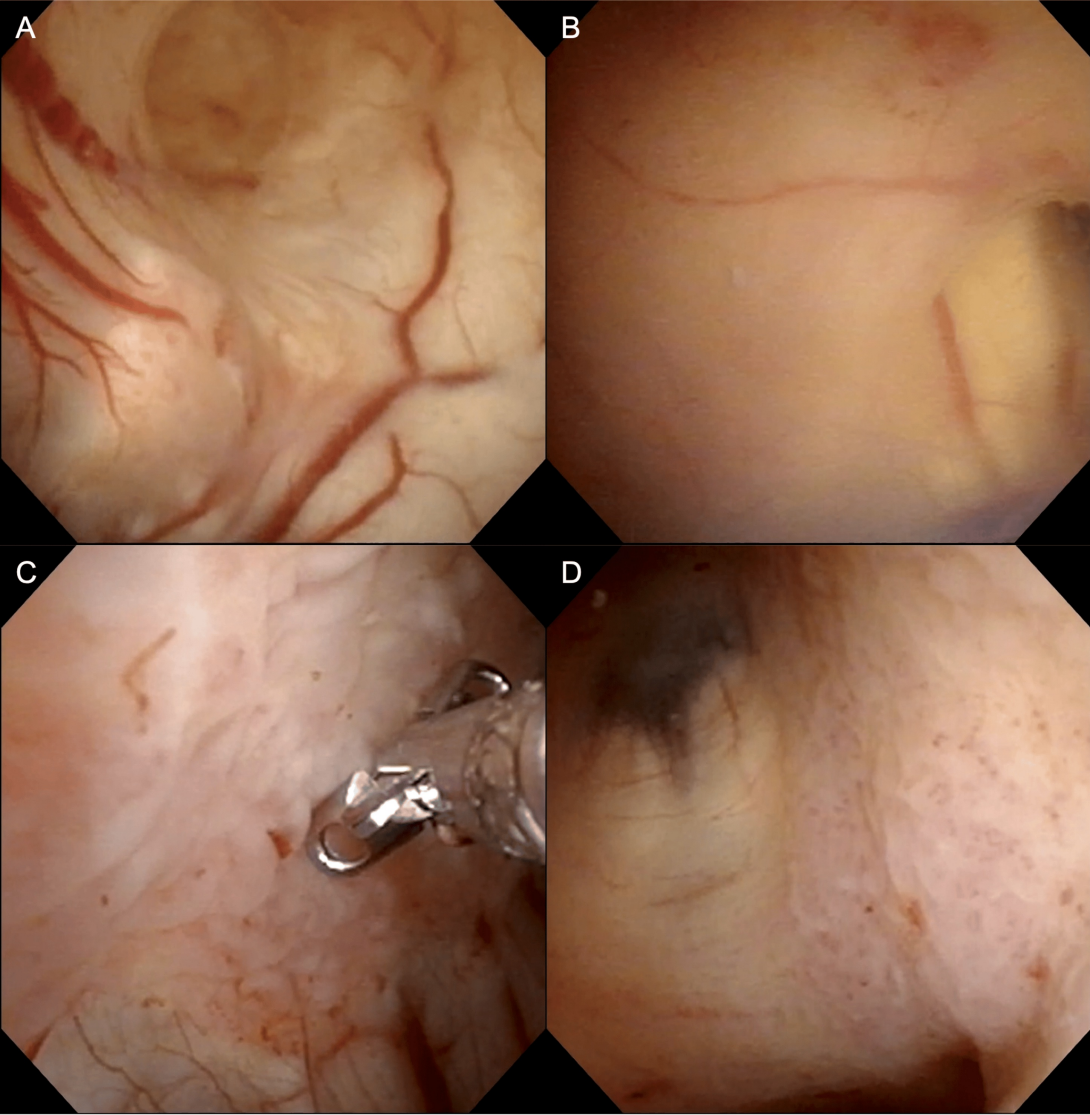
Endoscopic view of ependymal biopsy and third ventriculostomy. The ependyma of the right lateral ventricle was broadly covered with membranous structures. (A) Region near the foramen of Monro. (B) The body of the lateral ventricle. Part of the lesion had an appearance resembling fish eggs, and some of the specimens were biopsied from the medial wall of the right lateral ventricular body (C). The third ventricle was also affected by membranous and fish-egg-like lesions (D)

To diagnose and treat the hydrocephalus with minimal invasiveness, an endoscopic ependymal biopsy combined with a third ventriculostomy was performed on the same day. Under general anesthesia, a flexible neuroendoscope was inserted in the right lateral ventricle through a single burr hole in the right frontal region. At the time of endoscope insertion, the CSF was turbid, so observation was carried out while adequately perfusing with artificial CSF. The wall of the lateral ventricle was broadly covered by thin membranous structures within the entire observable range (Figures 2A, 2B). Part of the lesion had a fish-egg-like appearance and was biopsied (Figure 2C). The lesion affected even the third ventricle (Figure 2D), but no obstruction of the mesencephalic aqueduct was identified. Subsequently, a third ventriculostomy was performed. No significant abnormalities were detected in the tuber cinereum, which was perforated with forceps and dilated using a balloon catheter. The procedure enabled the bidirectional flow of CSF through the fenestration.

**Figure 3:**
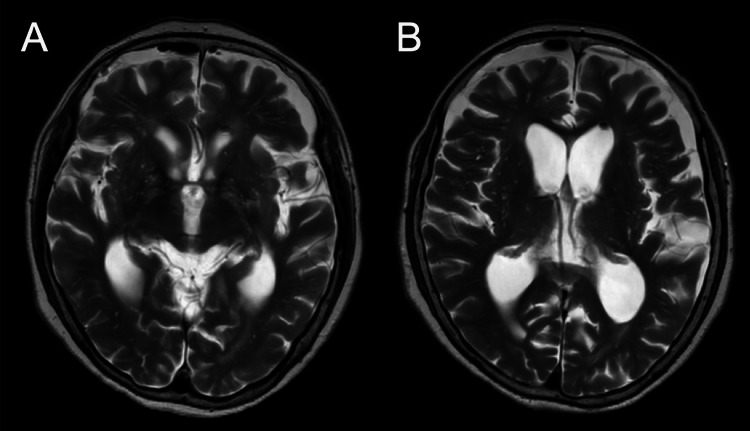
T2-weighted images obtained seven days after the surgical intervention. The ependyma of the right lateral ventricle was broadly covered with membranous structures. Sections through the third ventricle (A) and the lateral ventricles (B) Ventricular enlargement was alleviated

**Figure 4:**
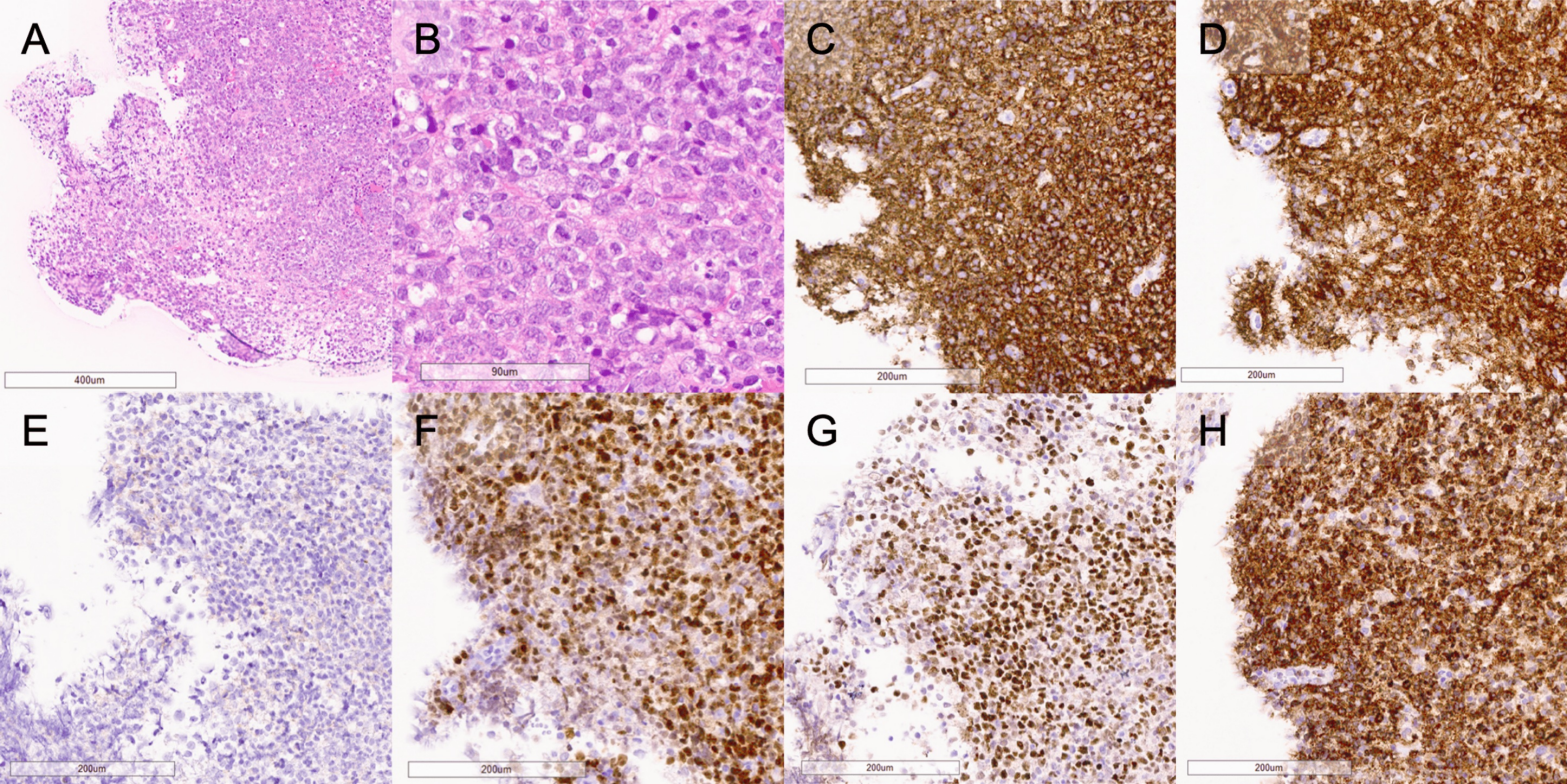
(A,B) Hematoxylin and eosin staining, showing diffuse and dense infiltration of atypical cells with large nuclei, prominent nucleoli, a high nucleocytoplasmic ratio, and poor cohesion. The magnification provided was 100× (A) and 400× (B). (C-H) Immunohistochemical staining revealed positivity for CD20 (C), CD79a (D), MUM1 (E), BCL-6 (F), and BCL-2 (G), and negativity for CD10 (H)

Postoperatively, the patient’s consciousness impairment improved, and she was able to resume eating and walking rehabilitation. Magnetic resonance images showed alleviation of the ventricle enlargement (Figures 3A, 3B). Tissue specimens exhibited diffuse and dense infiltration of atypical cells with large nuclei, prominent nucleoli, a high nucleocytoplasmic ratio, and poor cohesion (Figures 4A, 4B). Immunohistochemical staining revealed positivity for CD20, CD79a, MUM1, BCL-6, and BCL-2, and negativity for CD10, resulting in a diagnosis of primary diffuse large B-cell lymphoma of the central nervous system, specifically PLML (Figures 4C-4H). She was transferred to the Department of Hematology and began remission induction therapy, including high-dose methotrexate. However, her clinical condition did not improve further, and the remission induction therapy was discontinued after only one cycle. Tirabrutinib was then initiated, but she passed away three months after the start of remission induction therapy.

## Discussion

In this case, we could detect rapid clinical and radiological progression of communicating hydrocephalus, which allowed us to conduct a comprehensive evaluation, including contrast-enhanced MRI, considering both neoplastic and infectious diseases. These results enabled us to perform a biopsy alongside the treatment for hydrocephalus. However, without information on temporal changes, there could have been a risk of misdiagnosing the condition as idiopathic normal pressure hydrocephalus (iNPH), delaying the initial workup and leading to the placement of a conventional CSF shunt, and potentially missing the opportunity for a true diagnosis.

To the best of our knowledge, this is the first reported case of PLML manifesting as proportional hydrocephalus with normal pressure. Although 9%-33% of PLML cases are reported to involve ventricular enlargement, the chief complaints of those patients are mostly cranial nerve palsy or symptoms of increased intracranial pressure, such as headache or visual abnormalities [5,11]. On the contrary, it is extremely rare for PLML to present with nonspecific symptoms, including cognitive impairment or gait disturbance, as observed in this case. Nohira et al. reported a case of PLML manifesting with hydrocephalus, but that case showed disproportionate enlargement of the fourth ventricle, suggesting an obstructive mechanism below the fourth ventricle [12]. Ishizaki et al. described a case of PLML presenting as normal pressure hydrocephalus (NPH), but the diagnosis was based solely on clinical and radiological findings without pathological confirmation [13].

PLML is a very rare disease, and few systematic reports on this topic are available [5,11]. Additionally, the diverse presentation of the initial symptoms and nonspecificity of the radiological features often complicate the diagnostic process. The presenting signs and symptoms in order of frequency are as follows: cranial nerve palsy (58%), spinal symptoms (48%), headache (44%), ataxia (25%), and seizure (8%). In cases of cranial nerve palsy, cranial nerves VI (54%) and VII (29%) are most frequently affected, whereas spinal symptoms predominantly include leg weakness (74%) and bowel and bladder dysfunction (43%) [5]. Clinical manifestations depend on the distribution of dissemination, and it is uncommon for PLML with iNPH-like symptoms, including nonfocal symptoms like cognitive impairment or gait disturbance, as seen in this case. Regarding the radiological features, leptomeningeal enhancement is typical, although it has been documented that only 74% of cases demonstrate a positive result. The breakdown of this included cerebral convexities (38%), posterior fossa (29%), cranial nerves (29%), and spinal cord (79%) [5]. Regarding the enhancement of the ventricular wall, few cases have been reported, and the frequency remains unclear [12]. Of note, the positive rate of CSF cytology is 67%, and not a few cases show negative CSF cytology, which could make the preoperative diagnosis more difficult [5].

Common surgical approaches to treat hydrocephalus include CSF shunting and endoscopic third ventriculostomy (ETV). CSF shunting, particularly ventriculoperitoneal shunting (VPS), can be performed safely and effectively for both obstructive and communicating hydrocephalus, making it a widely utilized technique. However, in cases of hydrocephalus secondary to primary brain malignancies, including PCNSL, VPS may occasionally lead to tumor dissemination into the peritoneal cavity [14-16]. While ETV is generally employed for hydrocephalus caused by obstruction below the third ventricle, several studies have documented the effectiveness of ETV for communicating hydrocephalus, including iNPH, with reported success rates ranging from 21% to 100% [17-19]. In NPH cases, ETV is considered to break the vicious cycle of CSF stagnation within the ventricles, loss of parenchymal and vascular elasticity, and reduced pulsatile capacity of the brain by establishing systolic outflow from the third ventricle, thereby resolving the hydrocephalus. As for the present case, a biopsy of the ventricular lesions was also essential for pathological examination. The efficacy of the endoscopic approach is well documented, including its application to PCNSL [20,21]. To concurrently achieve a diagnostic biopsy and relieve the hydrocephalus, endoscopic management was selected. Whereas elective VPS was prepared in case of hydrocephalus correction failure, symptom alleviation was successfully achieved by ETV as expected. Furthermore, a definitive pathological diagnosis was established, enabling a seamless transition to appropriate chemotherapy. Not only in cases of PLML but also when addressing hydrocephalus with an unusual clinical trajectory or atypical imaging findings, the combined endoscopic approach of biopsy and third ventriculostomy may provide a potentially effective and reasonable treatment option.

## Conclusions

PLML is a rare variant of PCNSL, distinguished by the absence of parenchymal lesions. This report describes a rare case of PLML with iNPH-like symptoms and ventricular enlargement, yet demonstrating an extraordinarily rapid progression. Although this case is extremely rare, the possibility of PLML should be considered when evaluating hydrocephalus with an atypical course. For the diagnosis and resolution of atypical hydrocephalus, endoscopic biopsy combined with the third ventriculostomy can serve as an effective and reasonable treatment option.
